# Digital Gene-Expression Profiling Analysis of the Cholesterol-Lowering Effects of Alfalfa Saponin Extract on Laying Hens

**DOI:** 10.1371/journal.pone.0098578

**Published:** 2014-06-02

**Authors:** Lu Zhou, Yinghua Shi, Rui Guo, Minggen Liang, Xiaoyan Zhu, Chengzhang Wang

**Affiliations:** College of Animal Science and Veterinary Medicine, Henan Agricultural University, Zhengzhou, China; Clermont Université, France

## Abstract

**Background:**

To prevent cardiovascular disease, people are advised to limit their intake of dietary cholesterol to less than 300 mg/day. Egg consumption has been seriously reduced because of the high levels of cholesterol. The purpose of the present study was to evaluate the cholesterol-lowering effects of alfalfa saponin extract (ASE) in yolk and the molecular mechanisms underlying these effects using digital gene-expression profiling analysis. Liver and ovary tissues were isolated from laying hens fed with ASE for RNA sequencing.

**Results:**

The cholesterol content of the yolks of eggs from hens fed 120 mg/kg ASE declined considerably on day 60. Other groups (60, 240, 480 mg/kg ASE group) also showed decreases, but they were not significant. Digital gene expression generated over nine million reads per sample, producing expression data for least 12,384 genes. Among these genes, 110 genes showed greater than normal expression in the liver and 107 genes showed greater than normal expression in the ovary. Cholesterol 7 alpha-hydroxylase (*Cyp7a1*) and apolipoprotein H (*Apoh*), which act in the synthesis of bile acid and cholesterol efflux, showed more expression in the livers of hens given dietary ASE supplementation. In the ovary, levels of very low density lipoprotein receptor (*Vldlr*), apolipoprotein B (*Apob*), apovitellenin 1 (*ApovldlII*) and vitellogenin (*VtgI*, *VtgII* and *VtgIII*) in ovary decreased with dietary ASE supplementation.

**Conclusion:**

Transcriptome analysis revealed that the molecular mechanisms underlying the cholesterol-lowering effects of ASE were partially mediated by enhancement of cholesterol efflux in the liver and this reduced of cholesterol deposition in the ovary.

## Introduction

Cardiovascular disease is a major cause of death worldwide, especially in developed countries. Higher concentrations of circulating cholesterol are a major risk factor for cardiac problems. Limiting cholesterol intake to 300 mg/day can prevent elevated blood cholesterol levels and prevent increases in the risk of coronary heart diseases. This figure is given in both the National Cholesterol Education Program diet [1] and the American Heart Association dietary recommendations [2]. In developed countries, egg consumption has decreased considerably because the cholesterol, 200–300 mg per egg, can increase the risk of coronary heart disease [3], [4]. However, in China and or other developing countries, people usually eat eggs as their main source of dietary protein. Low-cholesterol eggs would be of great significance to public health. Ever since the 1970s, research efforts have been directed toward reducing egg cholesterol content in different ways. These have included genetic selection, altering the hens' diet with various nutrients and probiotics, and treating the hens with drugs, non-nutritive factors, and phytogenic extracts [5]–[8]. Consumers are likely to prefer eggs modified by phytogenic extract to eggs modified by drugs because they have fewer and less side effects.

Alfalfa saponin extract (ASE) is extracted from *Medicago sativa* L. The main active component is saponins. It also includes flavonoids and polysaccharides. The mixture of biologically active ingredients presented in ASE has pleiotropic physiological effects, including reduction of aphid population [9], [10], alteration nutrient digestion and fermentation in rumen [11], [12], antimicrobial activity [13], [14], and regulation of cholesterol and lipid metabolism [15]–[17]. Studies have been carried out to determine whether ASE can modulate cholesterol metabolism and decrease cholesterol levels in animals. Supplementing the diets of broilers with alfalfa significantly decreased total lipid and cholesterol content in poultry meat [18]. Broilers were supplied with a diet containing 0.06% polysavone (alfalfa extract) led to a decrease in abdominal fat weight at 5 and 6 week of age [19]. Cholesterol and triglycerides in the yolk, serum and liver were significantly reduced by dietary karaya saponins [20]. Karaya saponins also had the similar effect on Japanese laying quails [21]. Feeding the hens food containing 0.15% aqueous alfalfa extract caused the cholesterol and triglyceride levels in the yolks and the very low density lipoprotein cholesterol in serum and liver to decrease in laying hens [22]. Previous studies have also suggested the modulating effect of ASE on cholesterol metabolism in laying hens [23]. However, the underlying molecular mechanisms remain undefined.

More recently, RNA sequencing (RNA-seq) technology has been widely applied to evaluate specific, different, and abundant genes from millions of sequencing tags in experimental models [24]. This method can help us to clarify the cholesterol-lowering effect of ASE in laying hens. The liver is not only the major source of cholesterol biosynthesis *in vivo*, but also plays an important role in the regulation of cholesterol homeostasis. The ovary also plays an important role on cholesterol across the ovarian membranes and subsequent deposition in the developing yolk through receptor-mediated action. In this study, the liver and ovary tissues of laying hens were collected and digital gene expression (DGE) was performed to investigate the mechanisms underlying the cholesterol-lowering effect of ASE.

## Materials and Methods

### Ethics statement

All animal experiments were approved by the Animal Care and Use Committee of Henan Agricultural University. All animal work was performed according to regulations and guidelines established by the Ministry of Science and Technology of the People's Republic of China (Approval number: 2006–398). All efforts were made to minimize the suffering of the animals.

### Animals and grouping

A total of 150 Hy-Line Brown hens at the age of 27 weeks, weighing about 1.90 kg, the mean egg production of 90.51±2.24%, were randomly divided into five groups (five replicates were established per treatment, with 6 hens each replicate. The hens were fed in wire cages for 77 days (two hens per cage, measuring 480×380×340 mm (w×d×h)). The birds were provided with feed and water *ad libitum* and a photoperiod (16- h light/8- h dark) during the experimental period. The hens were kept at room temperature 25°C. None of the hens died or and drugs during this study.

Hens in each treatment were randomly fed five different experimental diets: Controls were fed a basal diet with 0 mg/kg ASE, and the other four groups were fed otherwise identical diets supplemented with 60 mg/kg, 120 mg/kg, 240 mg/kg, and 480 mg/kg ASE, respectively. The basal diet was designed to meet, or exceed, the National Research Council (NRC) nutrient requirements. The ingredients and calculated nutrient level of the basal diet are shown in Table 1. ASE was provided by Hebei Bao'en Biotechnology Co., Ltd (Shijiazhuang, China) as a standardized product that contained 61.64% saponins, 10.97% flavonoids, 8.12% polysaccharides, 7.11% moisture, and 12.16% unknown compounds.

**Table 1:** Composition and nutrient content of diet.

Ingredient	%	Nutrient level^2^	
Corn	69.70	Crude protein (%)	16.50
Soybean meal	14.45	Calcium (%)	3.44
Limestone power	8.50	Phosphorus (%)	0.59
Peruvian fishmeal	4.96	Available phosphorus (%)	0.42
Dicalcium phosphate	1.00	Lysine (%)	0.83
Salt	0.20	Methionine (%)	0.38
Choline chloride	0.10	Methionine + cysteine (%)	0.63
DL-methionine	0.09	Apparent metabolic energy (MJ/kg)	11.49
Trace mineral and vitamin premix^1^	1.00		

Note: ^1^Premix provided the following per kilogram of diet: 60 mg of iron, 80 mg of manganese, 8 mg of copper, 80 mg of zinc, 1 mg of iodine, 0.3 mg of selenium, 12,200 IU of vitamin A, 4125 IU of vitamin D_3_, 30 IU of vitamin E, 4.5 mg of vitamin K, 5 mg of vitamin B_12_, 2 mg of biotin, 5 mg of folic acid, 32.5 mg of niacin, 5.3 mg of pantothenic acid, 8 mg of pyridoxine, 8.5 mg of riboflavin, and 1 mg of thiamin.

2 Obtained by calculation.

### Samples preparation and analysis

Four eggs from each replicate were randomly collected over the course of 15 days and the cholesterol levels in the yolks were analyzed. Yolk samples were subjected to saponification and detection by high-performance liquid chromatography (HPLC) as described by Zhang *et al.* [25]. Two hens from each replicate were killed at the end of the experiment. The liver and ovary tissues were isolated, frozen immediately in liquid nitrogen and stored at −80°C until RNA extraction. According to the cholesterol levels in yolk, the control group and 120 mg/kg ASE group were chosen for digital gene-expression profiling analysis.

### DGE-tag profiling

#### RNA isolation and qualification

Total RNA was isolated using the Trizol reagent (Invitrogen, Code No.: 15596-018) according to the manufacturer's instructions. RNA degradation and contamination were monitored on 1% agarose gels. RNA was assessed for quantity and quality using a Nanodrop ND-1000 spectrophotometer and Agilent 2100 Bioanalyzer according to the manufacturer's instructions. Contaminating genomic DNA in total RNA samples were treated with DNase-I for later use.

#### Library construction and sequencing

At least 2 µg of total RNA (≥100 ng/µL) was pooled from ten tissues within each group: liver from the control group (Li_CK), liver from the 120 mg/kg ASE group (Li_Exp), ovaries from the control group (Ov_CK), ovaries from the 120 mg/kg ASE group (Ov_Exp), a total of four samples were used for library construction. Sequencing libraries were generated using NEBNext Ultra Directional RNA Library Prep Kit for Illumina (NEB) in accordance with the manufacturer's recommendations. After the clustering of the index-coded samples, each library preparation was sequenced on an Illumina HiSeq 2000 platform and 50 bp single-end (SE) reads were generated. The sequencing datasets have been uploaded to the NIH Short Read Archive, under accession number is SRP039463.

#### Mapping and analysis of DGE reads

For each DGE library, clean tags were obtained by filtering out adaptor sequences and low-quality tags. Then the clean tags were retained and mapped to the reference genome of *Gallus gallus* at ftp://ftp.ensembl.org/pub/release-72/fasta/gallus_gallus/dna by TopHat v1.4.0 [26]. TopHat was the selected as the mapping tool because it can generate a database of splice junctions based on the gene model annotation file and so create better mapping result than other non-splice mapping tools.

HTSeq v0.5.3 was used to count the reads mapped to each gene [27]. Then reads per kilobase transcriptome per million mapped reads (RPKM) of each gene were calculated based on the length of the gene and reads count mapped to this gene. RPKM considers the effect of sequencing depth and gene length for the reads count simultaneously. It is currently the most common method of for estimating levels of gene expression [28]. In order to balance the number of false positives and false negatives a RPKM threshold value of 0.3 was established to determine whether or not given genes was expressed, as in several other studies [29]–[31].

Because there were no biological replicates, for each sequenced library, the read counts were adjusted by edgeR package through one scaling normalized factor [32]. Differential expression analysis of two conditions was performed using the DEGSeq R package v1.12.0 [33]. The *P*-values were adjusted using the Benjamini and Hochberg method [34]. Corrected *P*-values are also called q-values. The q-values of 0.05 and log2 (Fold_change) with no limitations served as the threshold of significance for differential expression.

#### Functional analysis of differentially expressed genes

Gene ontology (GO) enrichment analysis of differentially expressed genes was implemented using GOseq [35], in which gene length bias was corrected. GO terms with q-values less than 0.05 were considered significantly enriched by differential expressed genes.

Kyoto Encyclopedia of Genes and Genomes (KEGG) is a database resource used to facilitate understanding of the high-level functions and uses of the biological system (http://www.genome.jp/kegg/). Here, KOBAS software was used to test the statistical enrichment of differential expression genes in KEGG pathways [36].

### Quantitative real-time PCR validation

This validation was performed by using RNA from eight individuals per group, and they were also treated with DNase-I. cDNA was synthesized with Reverse Transcriptase M-MLV (RNase H-) (TaKaRa, Code No.: D2639A) using the oligo dT primer. Quantitative real-time PCR (qRT-PCR) was performed on selected genes known to have a relationship with cholesterol metabolism. The Chicken lamin B receptor (*Lbr*) served as an internal control gene [37]. Primer sequences were designed using Primer 5 (ref. Table S1). qRT-PCR with the SYBR Real-time PCR Premix Ex TaqTM (Tli RNaseH Plus) (TaKaRa, Code No.:RR420A) was carried out on a Mastercycler ep realplex Real-Time PCR System (Eppendorf, Germany). The results were normalized to the expression level of the constitutive *Lbr*. A relative quantitative method (2^−ΔΔCt^) was used to evaluate the quantitative variation.

### Statistical analysis

All results are expressed as mean ± SD. The data were evaluated by one-way ANOVA (SPSS for Windows, version 19.0), and the differences between the means were assessed using Duncan's test. *P*<0.05 was considered statistically significant. DGE results were analyzed using R software (see materials and methods for software and other details).

## Results

### ASE and laying performance

Performance data of laying hens fed with different levels of ASE are presented in Table 2. These effects of ASE were apparent during the experimental periods. ASE hens all ate significantly less than in controls (*P*<0.05). Hens fed 240 mg/kg ASE produced observably smaller egg than control hens (*P*<0.05). However, ASE had no significant effect on the number of eggs produced (*P*>0.05). Unlike in controls the ratio of feed efficiency all had a reduction, but it didn't reach a significant level (*P*>0.05).

**Table 2:** ASE and laying performance.

Item	ASE in diet (mg/kg)				
	0	60	120	240	480
Feed intake (g/hen)	122.07±2.47^a^	116.18±5.33^b^	113.94±2.93^b^	114.53±2.58^b^	116.68±2.47^b^
Egg production (%)	89.82±1.46^ab^	91.02±1.19^b^	90.56±1.10^b^	90.60±2.61^b^	87.75±1.50^a^
Egg mass (g)	62.06±1.28^a^	62.38±0.88^a^	61.59±0.67^ab^	60.57±0.80^b^	62.81±0.62^a^
Feed efficiency (feed/egg)	2.18±0.10	2.03±0.11	2.04±0.08	2.09±0.11	2.11±0.07

a,b Means in the same row not sharing a common superscript differ significantly at *P*<0.05.

### ASE and cholesterol in yolks

The effect of different concentrations of ASE on cholesterol concentration in yolk is shown in Table 3. Conclusively, the cholesterol concentration in the yolks produced by hens in the ASE-supplemented tended to be lower than in the control group throughout the experimental period. On day 15, the concentration of yolk cholesterol in four treatment groups decreased, but this decrease was not significant (*P*>0.05). However, on day 30, the yolk cholesterol concentration was significantly lowered in all treatment groups than in the control group (*P<*0.05). On day 45, in the groups fed 120 mg/kg and 240 mg/kg ASE, yolk cholesterol concentrations were also lower than in the control group (*P<*0.05). The cholesterol concentration in yolks derived from the 120 mg/kg ASE group had obviously declined by day 60 (*P<*0.05).

**Table 3:** Dietary ASE and concentrations of yolk cholesterol (mg/g).

Parameter	ASE in diet(mg/kg)				
Period	0	60	120	240	480
Day 15	8.89±0.21	8.20±0.41	8.27±0.07	8.46±0.31	8.49±0.19
Day 30	9.23±0.24^a^	8.22±0.25^b^	8.35±0.59^b^	8.49±0.61^b^	8.49±0.13^b^
Day 45	9.33±0.24^a^	9.09±0.26^ab^	8.78±0.32^b^	8.87±0.11^b^	9.02±0.46^ab^
Day 60	9.57±0.41^a^	9.37±0.25^ab^	8.89±0.45^b^	9.47±0.39^ab^	9.31±0.36^ab^

a,b Means in the same row not sharing a common superscript differ significantly at *P*<0.05.

### Digital gene expression sequencing

In order to investigate the transcriptome response to cholesterol-lowering effect of ASE, RNA-seq of Li_CK, Li_Exp, Ov_CK and Ov_Exp libraries were performed using Illumina sequencing platform. The major characteristics of four libraries are summarized in Table 4. The Li_CK, Li_Exp, Ov_CK and Ov_Exp libraries were found to contain 14,860,639, 9,687,839, 12,617,106 and 11,261,644 short reads, respectively. After filtering out the adaptor tags and low-quality tags, 14,749,006, 9,602,093, 12,498,438 and 11,147,348 clean reads were retained, respectively, from which 6,521,091, 4,618,264, 8,716,000, and 7,781,418 unique reads were obtained.

**Table 4:** Basic characteristics of tags in four libraries and data of sequencing reads mapping to the reference genome.

Sample name	Li_CK	Li_Exp	Ov_CK	Ov_Exp
Raw reads	14,860,639	9,687,839	12,617,106	11,261,644
Clean reads	14,749,006	9,602,093	12,498,438	11,147,348
Unique reads	6,521,091	4,618,264	8,716,000	7,781,418
Duplication rate (%)	55.79	51.90	30.26	30.19
Q30 (%)	97.07	97.08	96.72	96.75
GC content (%)	49.30	49.27	50.52	50.37
Total mapped	11,686,971 (79.24%)	7,497,029 (78.08%)	9,456,291 (75.66%)	8,416,881 (75.51%)
Uniquely mapped	11,533,304 (78.20%)	7,397,719 (77.04%)	9,244,206 (73.96%)	8,212,214 (73.67%)
Reads map to '+'	5,793,756 (39.28%)	3,718,254 (38.72%)	4,622,787 (36.99%)	4,108,130 (36.85%)
Reads map to '-'	5,739,548 (38.91%)	3,679,465 (38.32%)	4,621,419 (36.98%)	4,104,084 (36.82%)
Non-splice reads	9,070,670 (61.50%)	5,845,095 (60.87%)	7,602,596 (60.83%)	6,766,852 (60.70%)
Splice reads	2,462,634 (16.70%)	1,552,624 (16.17%)	1,641,610 (13.13%)	1,445,362 (12.97%)

Note: 1. “+” refers to sense strands, “−” refers to anti-sense strands.

2. “Non-splice reads” refers to reads for the entire sequence is mapped to one exon; “Splice reads” also called junction reads, refers to reads mapped to the border of exon.

In order to control the quality of libraries, the error rate and GC content of the sequences were inspected (Table 4). The error rate distribution and GC distribution were also configured (ref. Figure S1 and S2). The inspection of transcript homogeneity was performed to confirm that there was no problem in the process of library construction (ref. Figure S3). These findings confirmed that the quality of four libraries was excellent.

### Mapping reads to the transcriptome

To profile the gene expression, the sequencing reads in four libraries to *Gallus gallus* genome were compared to TopHat v1.4.0. The results shown in Table 4 indicate that a substantial proportion of clean tags (79.24%, 78.08%, 75.66% and 75.51% in Li_CK, Li_Exp, Ov_CK and Ov_Exp libraries, respectively) matched the *Gallus gallus* genome. Some of these tags could be uniquely mapped to the reference genome, 11,533,304 (78.2%) and 7,397,719 (77.04%) tags in hepatic libraries and 9,244,206 (73.96%) and 8,212,214 (73.67%) tags in ovarian libraries. More than 60% of clean reads were non-splice reads in every sample. Then 12,824, 12,384, 14,424 and 14,284 genes were found to be expressed in four libraries, respectively.

**Figure 1:**
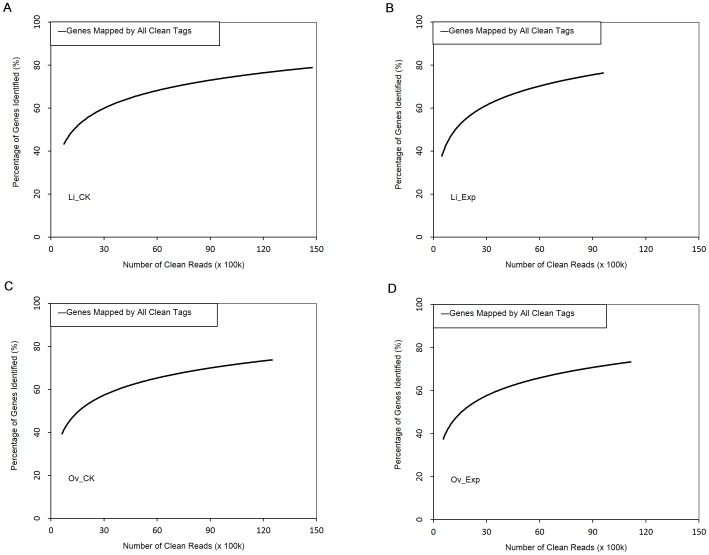
Saturation curve analysis of the digital gene expression tag libraries generated from four samples. A–D denote Li_CK, Li_Exp, Ov_CK and Ov_Exp, respectively. The relative number of genes identified (y axis) increased as the total tag number of tags (x axis) increased.

Sequencing data saturation analysis was performed to confirm whether the expression levels of detected genes increased proportionally to the total number of clean reads. Figure 1 showed that all the four libraries could reflect a transcriptomic profiling of ASE in the liver and ovaries of laying hens. The number of detected genes plateaued when it approached 4 million.

Statistical analysis of the total mapped reads in Li_CK, Li_Exp, Ov_CK and Ov_Exp libraries was performed to compare different parts of the reference genome. In two hepatic libraries, the relative number of reads mapped to exon was no less than 80%. However, in two ovarian libraries, the relative numbers were about 70% (ref. Figure S4). This might be because these gene annotation libraries are less complete. The distribution and density of total mapped reads in *Gallus gallus* chromosomes was also determined (ref. Figure S5). This analysis may facilitate the identification of chromosomes with extraordinary transcriptional activity after ASE supplementation.

### Analysis of gene differential expression

For analysis of gene expression, the number of unambiguous clean tags for each gene was calculated and normalized to RPKM. A RNA-Seq correlation test and RPKM distribution of all genes were performed to check the variation of CK samples and Exp samples in two tissues. In liver and ovary samples, the Pearson's correlation coefficients were 0.978 and 0.975, respectively. RPKM distribution and RPKM density distribution showed there to be few different genes in the same tissues (ref. Figure S6).

These results above indicated that there may not be many differentially expressed genes in in Li_CK, Li_Exp, Ov_CK and Ov_Exp libraries. A loose transcript detected with q-value <0.05 and log2 (Fold_change) with no limitations was used to compare the experimental library to the control library in two different tissues. Here, 110 significantly expressed genes were identified in liver samples; 66 were up-regulated and 44 were down-regulated. In ovary samples, 107 significantly expressed genes were obtained; 63 were up-regulated and 44 were down-regulated (ref. Table S2).

### Functional annotation of differentially expressed genes

**Figure 2:**
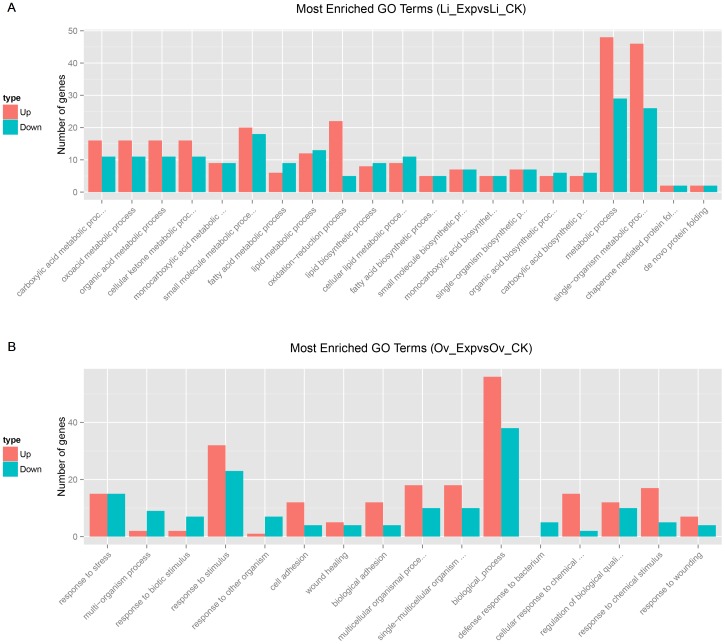
GO functional enrichment of genes differentially expressed in the liver and ovary. Gene classification based on gene ontology (GO) for differentially expressed genes in the liver and ovaries of laying hens. The number of genes in GO terms was analyzed using GO Slim Assignment. The *y-axis* and *x-axis* indicate the number of genes in each cluster and the names of clusters, respectively. Only biological processes were used for GO analysis.

GO functional enrichment analysis was performed to characterize the functional consequences of changes in gene expression associated with ASE. According to the GO enrichment, the genes in liver related to fatty acid biosynthetic, fatty acid metabolic, lipid biosynthetic, and lipid metabolic processes were common among the differentially expressed genes (Figure 2A). Most of the gene sets demonstrated regulated expression in the ovary tissue library, and most sets were associated with biological processes such as response to stimulus and stress (Figure 2B).

KEGG pathway analysis of differently expressed genes was also performed. The classifications indicated that there were some genes for which the expression intensity changed in relation to fatty acid metabolism, glycolysis/gluconeogenesis, the peroxisome proliferator-activated receptors (PPAR) signaling pathway, and other pathways in hepatic tissue (ref. Table S3). In addition, the ECM (the extracellular matrix)-receptor interaction, lysosome and focal adhesion were enriched among genes that were differentially expressed in ovarian tissue.

### Confirmation of differential genes by qRT-PCR

**Figure 3:**
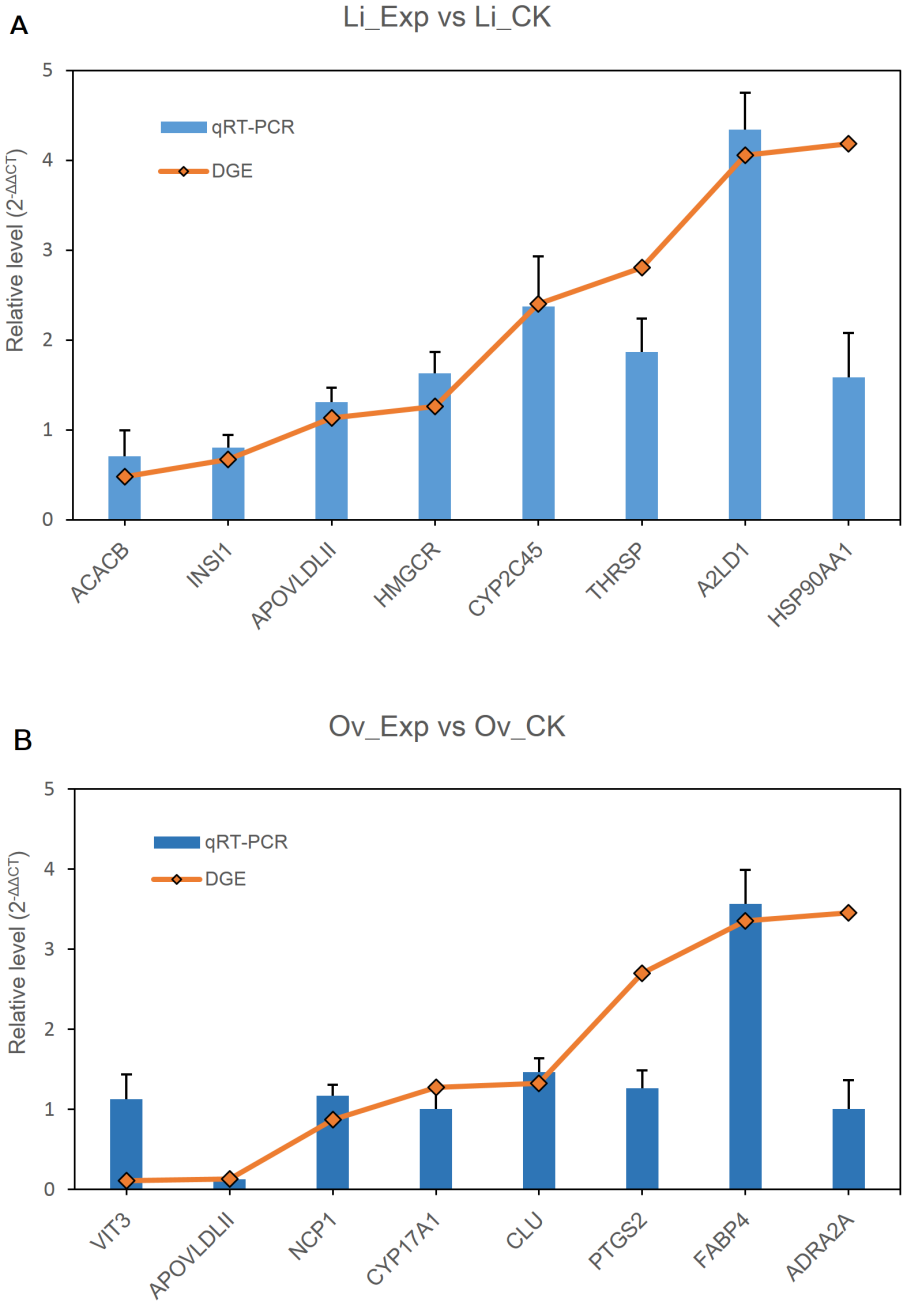
Sequencing and qRT-PCR. Quantitative RT-PCR validation of differentially expressed genes in control (a basal diet) and treatment (diet with 120 mg/kg ASE) samples from liver and ovary tissues, including eight genes. All data were normalized to the expression level of actin. Data represent fold change of relative quantification in treatment vs. control samples. The error bars represent the range of the fold change as determined by the data assist software.

To examine the results of tag-mapped genes, eight genes from the liver and eight genes from the ovary were selected for qRT-PCR and used to validate the concordance of fold change. The expression of eleven genes (*Acacb*, *InsI1*, *ApovldlII*, *Hmgcr*, *Cyp2c45*, and *A2ld1* in liver tissue; and *ApovldlII*, *Ncp1*, *Cyp17a1*, *Clu* and *Fabp4* in ovarian tissue), as examined by qRT-PCR, was consistent with patterns of Tag-mapped analysis (ref. Figure 3). Three genes (*Thrsp*, *Hsp90aa1*, and *Ptgs2*) behaved similarly between qRT-PCR and RNA-seq method. As shown in Figure 3 there were only two genes (*Vit3* and *Adra2a*) that did not show consistent expression between qRT-PCR and Tag-mapped data sets. These data added more weight to the credibility of the DGE results in this study.

## Discussion

### ASE, laying performance, and yolk cholesterol level

The present results suggest that the poor palatability of ASE diet due to the bitterness of saponin might be the primary reason behind lack of appetite. The findings were also consistent with those of a previous study on laying hens fed dietary saponin [38]. Except in the 240 mg/kg ASE group, egg mass showed no significant differences from controls. This may have caused the decline in feed intake. None of the ASE treatment groups had any appreciable differences from controls with respect to egg production or feed efficiency. Deng *et al.* also showed that alfalfa extract had no significant effect on the laying performance of hens [22].

The results of the present study, clearly confirmed that diets supplemented with 120 mg/kg ASE had a cholesterol-lowering effect on the yolks of laying hens. ASE at 120 mg/kg significantly reduced yolk cholesterol concentration by day 60. However, the 240 and 480 mg/kg groups showed no significant differences. The reasons underlying this phenomenon are not well established, but there are two possible factors: a) doses of 240 and 480 mg/kg ASE are too high for laying hens to take up or make full use of, while a dose of 120 mg/kg ASE may be exactly the right amount to produce a cholesterol-lowering effect. b) The concentration of dietary ASE may be too high, which results in cholesterol-related metabolic disorders in hens. The reduction in concentrations of cholesterol in egg yolks observed in the present study is similar to the 11.7% reduction in yolk cholesterol reported by Deng *et al.* for hens fed on 0.15% aqueous alfalfa extract supplemented diet [22]. As a result of yucca-saponins supplementation, yolk cholesterol concentration tended to decline by 11.5% [39]. However, a previous study showed that dietary alfalfa at varying saponin extract concentrations had no effect on yolk cholesterol levels [40]. This discrepancy, however, might be due to the different saponins ingested, different chicken varieties, age of hens, or other factors. In the present study, supplementing 120 mg/kg ASE played a positive role in lowering yolk cholesterol content. Based on this result, the group with 120 mg/kg ASE was used for digital gene-expression profiling analysis.

### Data analysis and verification for DGE

To clarify the molecular mechanisms underlying these processes, transcriptome sequencing was used on two different types of tissues (i.e., liver and ovary) from control group and 120 mg/kg ASE group. Until now, no study has explored the mechanisms underlying the cholesterol-lowering effects of ASE using genome-wide expression analysis. In the present study, a direct digital readout of tags and achieved an essentially dynamic range of genes from the libraries. In this way, the present study is the most comprehensive analysis of the cholesterol-lowering effects of the transcriptome. A mean of 11,999,221 clean reads were obtained per library (Table 4). This was more than enough to deliver sufficient sequence coverage for transcriptome profiling. Unfortunately, only 110 genes in the liver and 107 genes in the ovary were identified out of approximately 12,604 and 14,354 tag-mapped genes, respectively. The reasons might lead to this results: a) Reads were from 3'UTR of the mRNAs or precursor mRNA; b) Gene annotation of *Gallus gallus* were incomplete, and the reads would not be annotated to genes if there was a 2-bp mismatch; c) A genetic variation between *Gallus gallus* and layers contributed the slightness of the differences in genes [41]. In this way, only a low proportion of differentially expressed reads could be matched to annotated genes. Fortunately, some genes modified in bile acid metabolism in the liver and cholesterol deposition in the ovary were identified, indicating the cholesterol-lowering effects of ASE.

To validate the DGE method, the levels of 16 genes were confirmed by qRT-PCR. DGE results were compared to qRT-PCR results, and the trends of up-regulation and down-regulation were found to be similar. *Vit3* and *Adra2a* did not match the magnitude of the DGE results. The expression of two genes was low, so it was so difficult to use qRT-PCR to produce an accurate estimation.

### Differentially expressed genes with GO and KEGG annotation in the liver

According to the GO classification, some gene sets demonstrated downregulated expression in the hepatic tissue library of laying hens; and were associated with lipid metabolism. In a comparison between GO classification and the KEGG pathway analysis, the same genes were found in different pathway in the liver. The expression of genes regulated to fatty acid synthesis increased with supplementation with 120 mg/kg ASE; for example, *Acaca* and *Fasn* were significantly upregulated. However, the expression levels of *Acacb*, the other *Acac* isoform, were lower than in the control group. In fatty acid metabolism pathways, six liver genes associated with the degradation of fatty acids were markedly down-regulated by ASE supplementation. These included *Cpt1a*, *Acox1*, *Ehhadh*, *Acaa1*, *Hadhb*, and *Peci*. Functional analysis also showed genes to be differentially expressed in the glycolytic pathway in the liver after ASE supplementation. Eight of these genes were significantly upregulated, *Pgm1*, *Pgk1*, *Ldha*, *Aldh9a1*, *Pck1*, *Gapdh*, *Adh-1*, and *Aldh2*.

Expression of nine genes of the peroxisome proliferator-activated receptors (PPAR) signaling pathway was detected in the liver. It has been shown that PPARs play a regulatory role in the first steps of the reverse-cholesterol-transport pathway [42]. Most of genes in this pathway were dramatically downregulated after dietary ASE. These included *Apoa1*, *Acox1*, *Acaa1*, *Cpt1a*, *Ehhadh*, *Fabp3*, and *Lxrα*. The up-regulation of *Scd* and *Pck1* showed that ASE might have a positive effect on the monounsaturated fatty formation.

In this study, some genes were found to be associated with the cholesterol metabolism. These genes were not in any enrichment pathways. Two genes involved in the cholesterol biosynthesis, *Sc4mol* and *Cyp51a1*, were also significantly upregulated after ASE supplementation. Downregulated expression of *Insig1* indicated that ASE might have a positive effect on cholesterol biosynthesis through SREBP-mediated regulation [43].

These results suggest that ASE plays an unnecessary role in lowering the cholesterol levels of hens, but the truth is just the opposite. Cholesterol levels in yolks were markedly reduced. Phenomena in the liver may be the cause of this. First, laying hens usually meet their bodies' needs for cholesterol largely and entirely by *de novo* biosynthesis; they are generally fed little or no foods of animal origin. Second, there is a longstanding view that ASE can form insoluble complexes with cholesterol in the gut lumen [44]. In this way, the concentration of cholesterol in peripheral tissues may be too low to activate the reverse-cholesterol-transport pathway through the PPAR signaling pathway. Third, if ASE reduces the deposition of cholesterol in yolks (see below), then the organism might react as if it had a shortage of cholesterol and cholesterol biosynthesis may be triggered.

### Key genes adjusting cholesterol efflux in liver

It is here reported that the mRNA expression of both *Cyp7a1* and *Apoh* was significantly enhanced. The ***Cyp7a1*** gene encodes the enzyme cholesterol 7α-hydroxylase, which catalyzes the initial step of cholesterol catabolism and bile acid synthesis. *Cyp7a1* plays a critical role in whole body cholesterol homeostasis. With ASE supplementation, the upregulation of *Cyp7a1* increased the production of bile acids and reduced the level of cholesterol in hepatocytes. Similar findings were observed in rats treated with Korean red ginseng extract and platycodin D [45], [46]. ***Apoh*** encodes a single chain glycoprotein that has been implicated in the development of atherosclerosis. It has been reported that this glycoprotein can reduce cellular accumulation of cholesterol by decreasing cholesterol influx and increasing cholesterol efflux [47]. This suggests that *Apoh* may play an important role in the prevention of atherosclerosis. These findings indicate that the cholesterol-lowering effects of ASE in hepatic tissue were partially mediated by up-regulation of *Cyp7a1* and *Apoh*, which enhanced bile acid excretion.

### Function analysis of differentially expressed genes in ovary

To shed more light on the functional roles of differentially expressed genes responsible for the cholesterol-lowering effect of ASE, functional annotation was investigated using GO classification and KEGG pathway analysis between Ov_CK and Ov_Exp samples. The extracellular matrix (ECM) had a profound effect on cellular functions and probably played an important role in the processes of follicular development and atresia, ovulation, and development, maintenance, and regression of corpora lutea [48]. In the present study, irregular expression of genes in the EMC-receptor interaction pathway suggested that ovarian tissues might be involved in different remodeling cycles. Because focal adhesions serve as the mechanical linkages to the ECM, these differentially expressed genes were also obtained in a focal adhesion pathway.

Several upregulated genes known to be involved in the lysosome pathway were detected in ASE-treated tissues. Lysosomes can digest some cellular waste and other macromolecules are digested through phagocytosis, endocytosis, and autophagy. It also plays a role in the degradation of basement membrane, which causes the follicles to rupture and release mature eggs. These results suggest that dietary ASE might have an effect on the acceleration of ovulation in laying hens.

### Cholesterol deposition in yolk and its regulation in ovary

There is minimal cholesterol in a conventional laying diet. Hens *de novo* synthesize most of cholesterol needed for eggs, structural components of cell membranes, the precursors to sex and adrenal hormones, vitamin D_3_, and bile acids. In hens, after biosynthesis in the liver, cholesterol is incorporated into triglyceride-rich very-low-density lipoprotein (yolk targeted VLDL, also called VLDLy) and vitellogenin (VTG) particles. As the hens sexually mature, massive amounts of VLDLy particles and VTG, including cholesterol, are produced and secreted into plasma by liver under the stimulation of estrogen [49]. In addition to apoB, apolipoprotein VLDLII (apoVLDLII) also play a role in the assembly and secretion of the VLDLy particles. Then the VLDLy particles escape through lipolysis. Through VTG transport, they move from the plasma to the ovary, where they are taken up into the developing follicles through receptor-mediated endocytosis. VLDLy and VTG constitute 95% and 5% of the cholesterol in yolk, respectively [50], [51].

In this study, ASE was found to positively regulate the expression of key genes involved in cholesterol transportation in the ovary. ASE had a modest effect on the downregulation of mRNA levels of ***Vldlr***, a cell-specific receptor that mediates the endocytosis of VLDLy and VTG [52]. Because, the biological function of yolk is to provide various nutrients to the embryo, like lipids, proteins, fat-soluble vitamins, and other substances essential to the growth and development of the embryo, the ovarian genes in this study could not change greatly without resulting in embryo death. The slight downregulation of *Vldlr* expression caused a considerable reduction in the level of cholesterol in yolk.

ApoB and apoVLDLII are components of VLDLy particles. These particles transport cholesterol and triglyceride from the liver to the ovary for deposition in yolk. ***Apob*** and ***ApovldlII*** showed markedly less expression in the ASE group than in the control group. These changes, which appear in the transposition of VLDLy, decreased cholesterol deposition in yolk.

Chicken **vitellogenin** consists of three species designated *VtgI*, *VtgII* and *VtgIII*. All three genes were found to be expressed at lower level in dietary ASE than in controls. *Vtg* makes up to 5% of the cholesterol in the yolks. This suggested that the deposition of cholesterol with VTG would be reduced in yolk.

Steroidogenic acute regulatory protein (***Star***) is a transport protein that facilitates transfer of cholesterol to the inner mitochondrial membrane. This is the rate-limiting step in the production of steroid hormones. In this study, the mRNA levels of *Star* were significantly higher in the ASE group than in the control group. Experimental analysis showed ASE to accelerate the actions of synthetic steroid, hormones and to contribute to cholesterol transformation. Cholesterol deposition in yolk decreased correspondingly.

In short, these findings in ovarian tissues echoed the decrease of cholesterol concentration in yolks. They also provide new information that may facilitate understanding of the molecular mechanisms underlying the cholesterol-lowering effects of ASE in egg yolk.

## Conclusions

Here, RNA sequencing was used to delineate global gene expression patterns in the liver and ovary tissues of laying hens with ASE treated. Using this method, the molecular events provide us with a new way of understanding the molecular mechanisms by which ASE reduces cholesterol in egg yolks. These results provide solid evidence on some functional roles of ASE in hepatic cholesterol efflux and deposition in ovaries, such as upregulation of *Cyp7a1* in the liver and downregulation of *Vldlr*, *Apob*, *ApovldlII* and *Vtg* in the ovary, which may be responsible for the cholesterol-lowering effects of ASE in yolks.

## Supporting Information

Figure S1 **Error distribution of the digital gene expression tag libraries generated from four samples.** A–D denote Li_CK, Li_Exp, Ov_CK and Ov_Exp, respectively. The error-rate (y axis) increased as sequencing reads (x axis) increased.
(TIF)

Figure S2 **GC content distribution of the digital gene expression tag libraries generated from four samples.** A–D denote Li_CK, Li_Exp, Ov_CK and Ov_Exp, respectively. GC content percent (y axis) distributed with sequencing reads (x axis) increased.
(TIF)

Figure S3 **Transcript homogeneity of the digital gene expression tag libraries generated from four samples.** A–D denote Li_CK, Li_Exp, Ov_CK and Ov_Exp, respectively. x-axis represent distance from 5′end of transcript(%), y-axis represent mean coverage.
(TIF)

Figure S4 **Distribution of four libraries reads in different regions of reference genome.** A–D denote Li_CK, Li_Exp, Ov_CK and Ov_Exp, respectively. Total mapped reads in different regions of *gallus gallus* genome.
(TIF)

Figure S5 **Distribution of total mapped reads density in** ***Gallus gallus*** **chromosomes.** A–D denote Li_CK, Li_Exp, Ov_CK and Ov_Exp, respectively.
(TIF)

Figure S6 **RPKM distribution and RPKM density distribution of different genes in the liver and ovary.** A: box-plot of RPKM. x-axis means sample names, y-axis means log_10_(RPKM). B: RPKM distribution. x-axis represent log_10_(RPKM), y-axis represent gene density.
(TIF)

Table S1 Primer sequences used for qRT-PCR.
(DOC)

Table S2 Genes differentially expressed in the liver and ovary in ASE and control groups.
(XLSX)

Table S3 KEGG pathway classification of differentially expressed genes in the liver and ovary.
(XLSX)
