# Supplementary figures and images for: Digital Gene-Expression Profiling Analysis of the Cholesterol-Lowering Effects of Alfalfa Saponin Extract on Laying Hens
Source: PLoS One. 2014 Jun 2;9(6):e98578. doi: 10.1371/journal.pone.0098578 (PMC4041749; doi:10.1371/journal.pone.0098578)

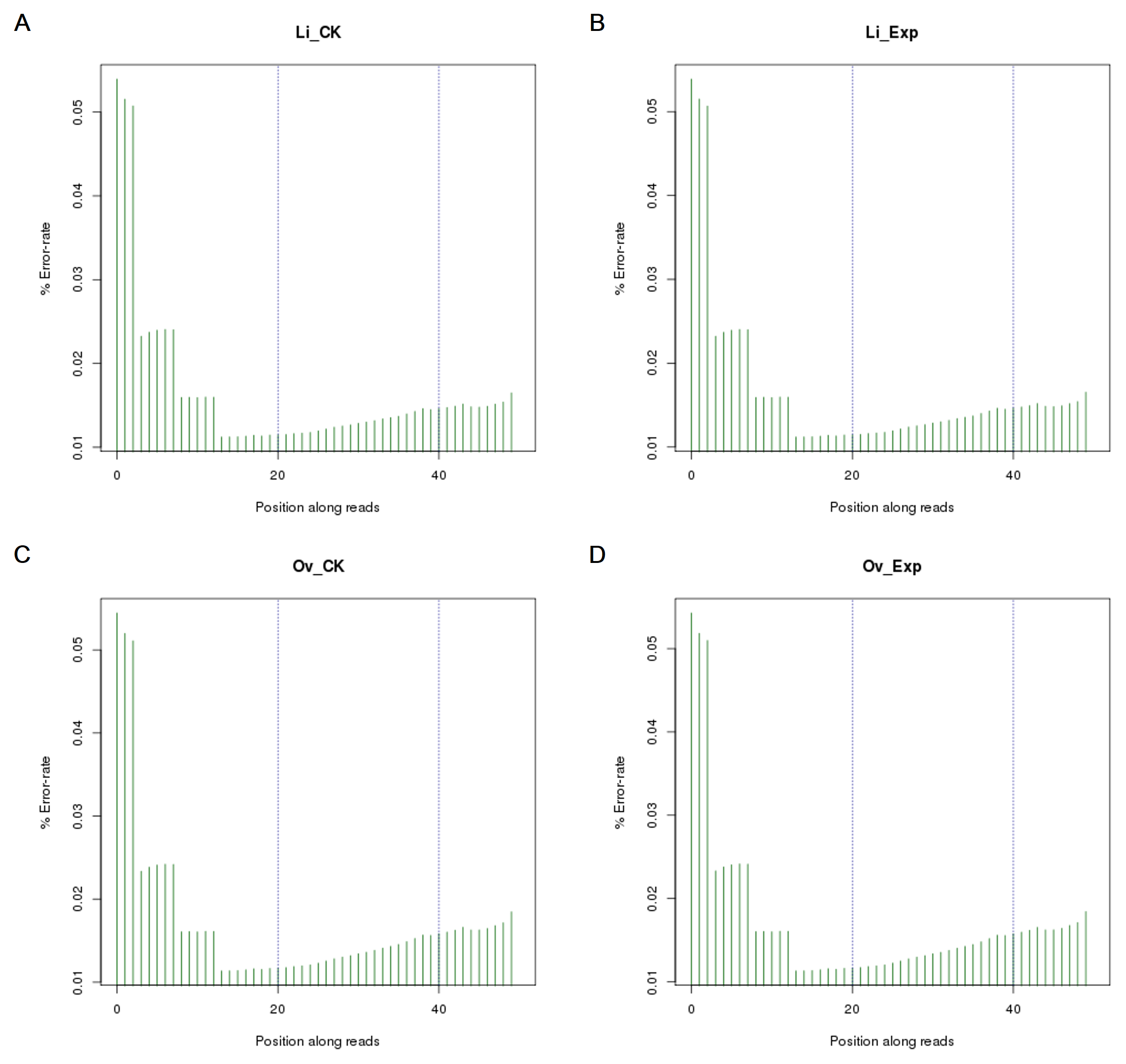

Supplement: Figure S1 — Error distribution of the digital gene expression tag libraries generated from four samples. A–D denote Li_CK, Li_Exp, Ov_CK and Ov_Exp, respectively. The error-rate (y axis) increased as sequencing reads (x axis) increased. (TIF) [file pone.0098578.s001.tif]

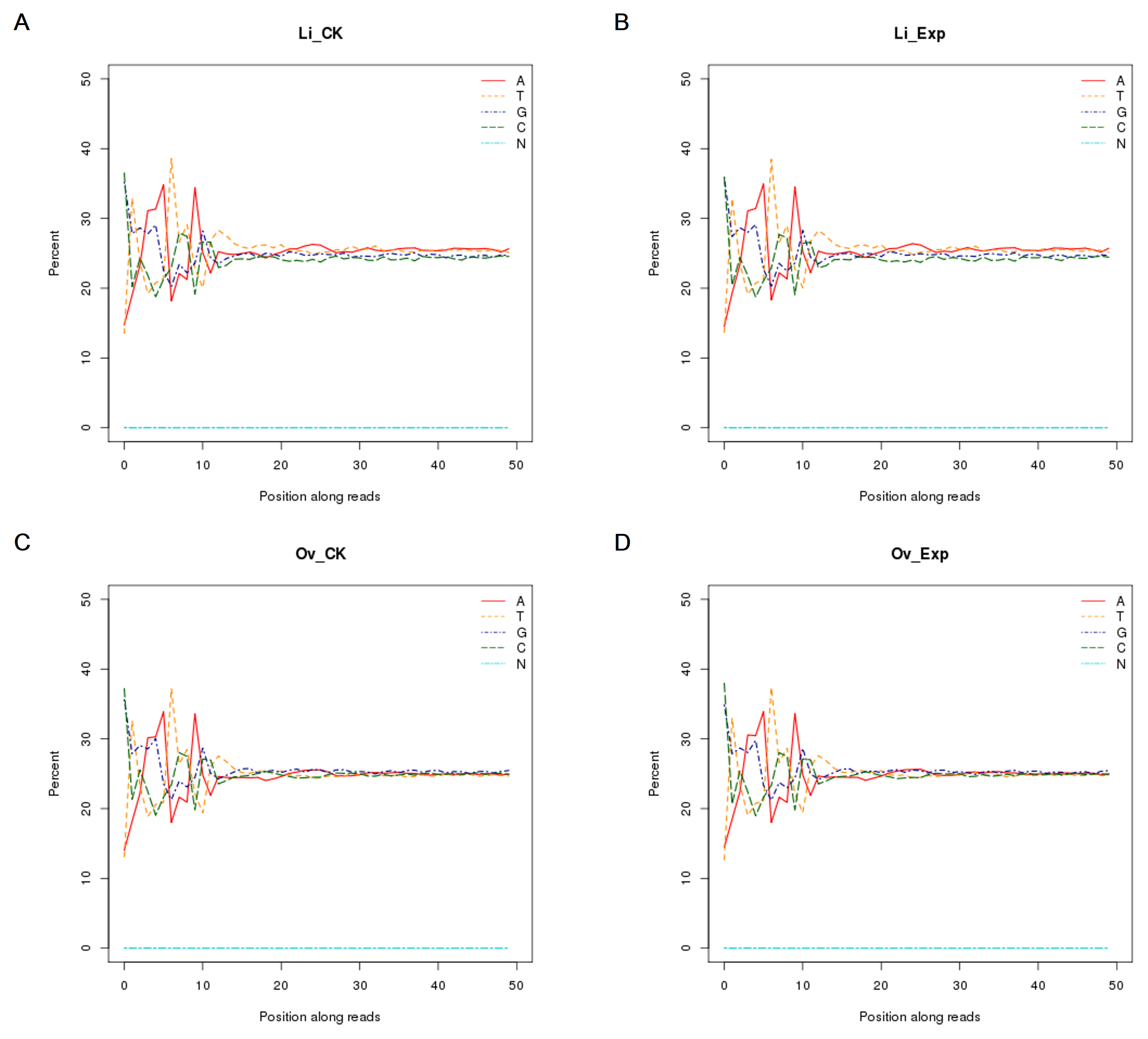

Supplement: Figure S2 — GC content distribution of the digital gene expression tag libraries generated from four samples. A–D denote Li_CK, Li_Exp, Ov_CK and Ov_Exp, respectively. GC content percent (y axis) distributed with sequencing reads (x axis) increased. (TIF) [file pone.0098578.s002.tif]

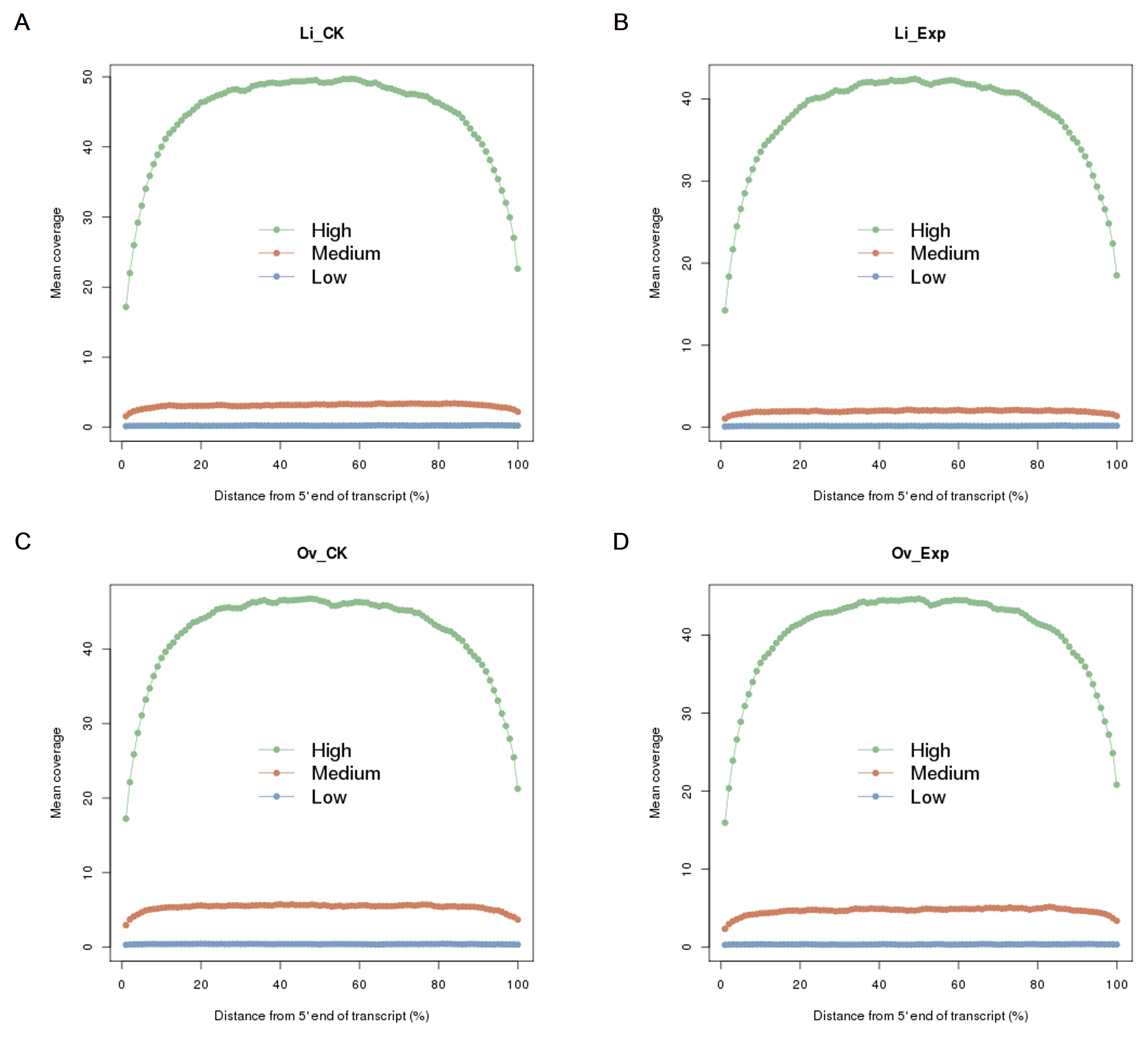

Supplement: Figure S3 — Transcript homogeneity of the digital gene expression tag libraries generated from four samples. A–D denote Li_CK, Li_Exp, Ov_CK and Ov_Exp, respectively. x-axis represent distance from 5′end of transcript(%), y-axis represent mean coverage. (TIF) [file pone.0098578.s003.tif]

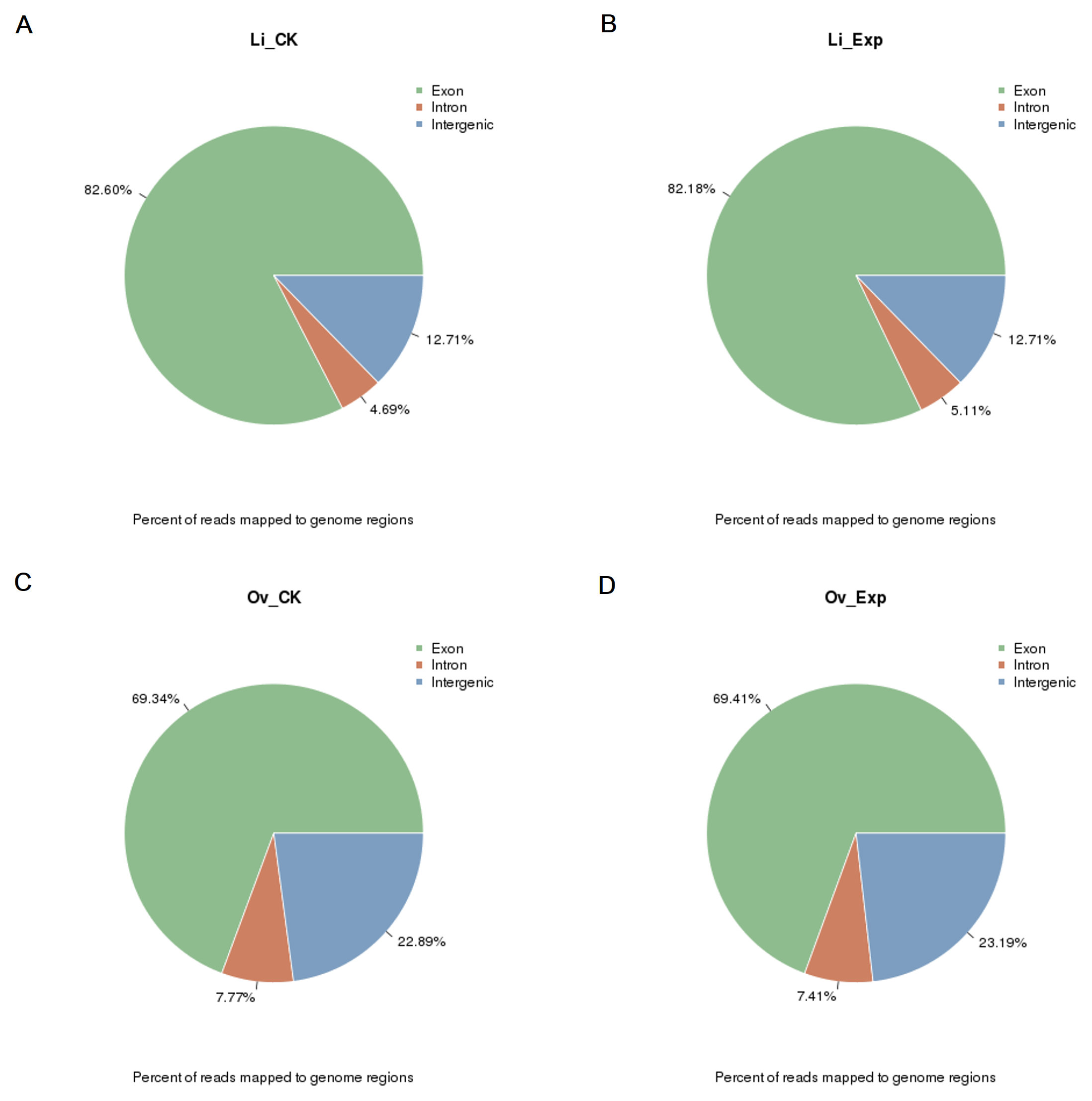

Supplement: Figure S4 — Distribution of four libraries reads in different regions of reference genome. A–D denote Li_CK, Li_Exp, Ov_CK and Ov_Exp, respectively. Total mapped reads in different regions of gallus gallus genome. (TIF) [file pone.0098578.s004.tif]

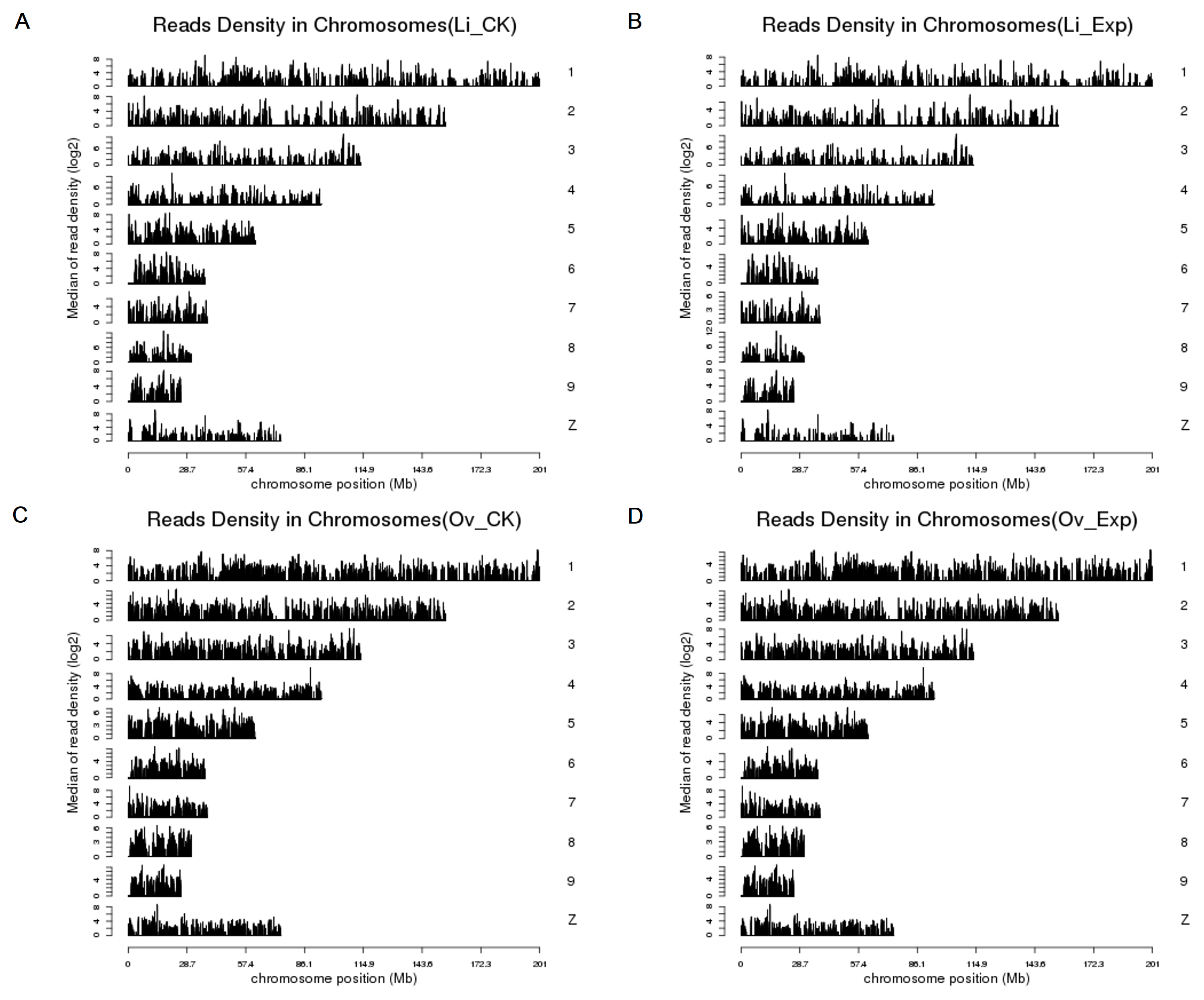

Supplement: Figure S5 — Distribution of total mapped reads density in Gallus gallus chromosomes. A–D denote Li_CK, Li_Exp, Ov_CK and Ov_Exp, respectively. (TIF) [file pone.0098578.s005.tif]

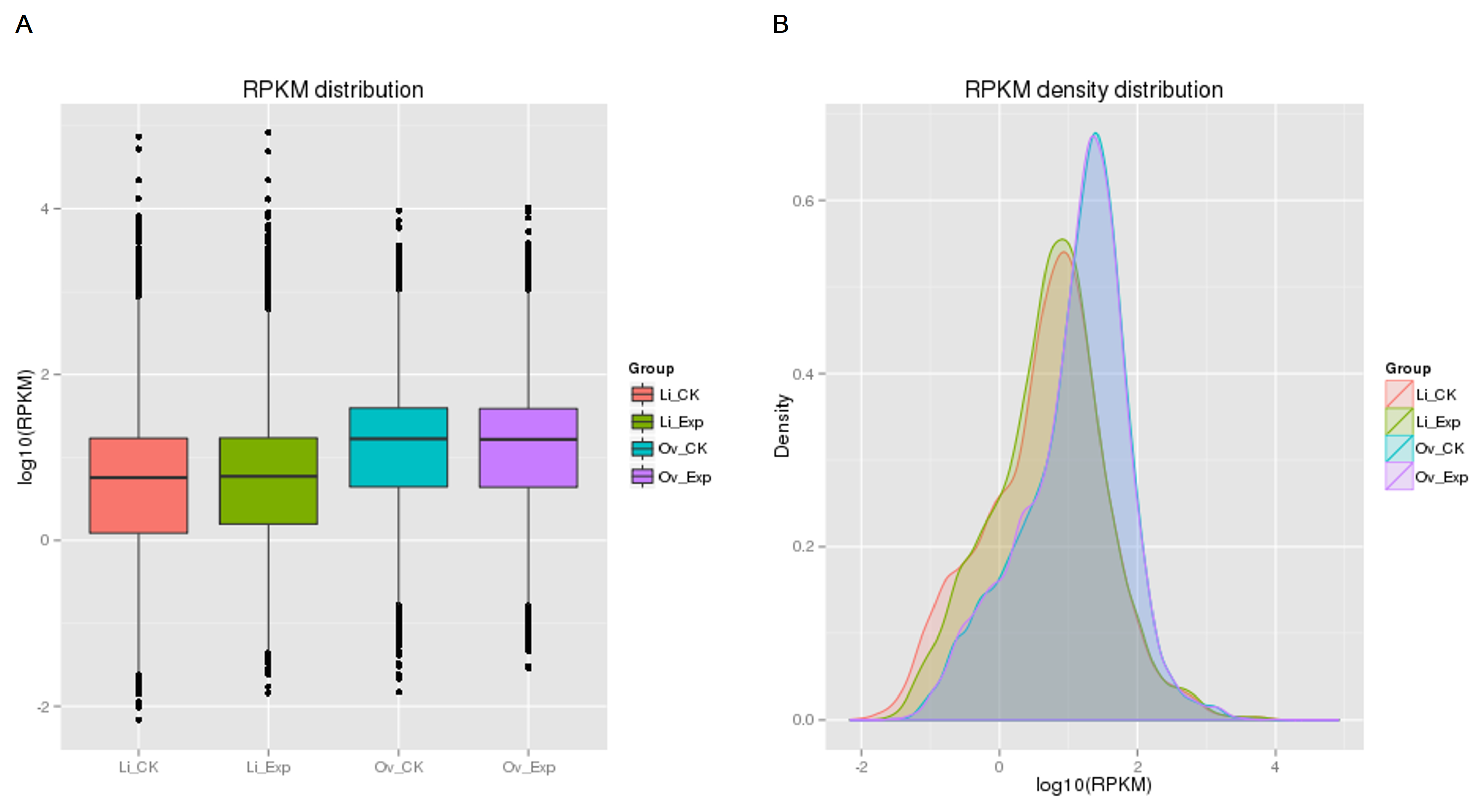

Supplement: Figure S6 — RPKM distribution and RPKM density distribution of different genes in the liver and ovary. A: box-plot of RPKM. x-axis means sample names, y-axis means log10(RPKM). B: RPKM distribution. x-axis represent log10(RPKM), y-axis represent gene density. (TIF) [file pone.0098578.s006.tif]
