# Supplementary material for: Digital Gene-Expression Profiling Analysis of the Cholesterol-Lowering Effects of Alfalfa Saponin Extract on Laying Hens
Source: PLoS One. 2014 Jun 2;9(6):e98578. doi: 10.1371/journal.pone.0098578 (PMC4041749; doi:10.1371/journal.pone.0098578)
Supplement: Table S1 — Primer sequences used for qRT-PCR. (DOC) [file pone.0098578.s007.doc]

Table S1. Primer sequences for qRT-PCR

| **Symbol** | **GenBank/Ensembl** | **Forward primer** | **Reverse primer** |
| --- | --- | --- | --- |
| LBR | NM_205342.1 | GGTGTGGGTTCCATTTGTCTACA | CTGCAACCGGCCAAGAAA |
| ACACB | XM_428114.4 | ATGAGATGTTCCGAAACGA | ATCTGCCATTCTGATGTACTCT |
| INSI1 | NM_001030966.1 | TCTTACCCTGATTTACTGC | CTGCCACTTAGCCTGAA |
| apoVLDL | NM_205483.1 | TTGGTGTAAAGGGCTGAACT | GGGACAGTGGTGCTAAGGAG |
| HMGCR | NM_204485.1 | TAGAGATAGGGACTGTTGGAG | TCACTGTAGCACACACGATT |
| CYP2C45 | NM_001001752.1 | GCACCTTTAGGAAGAGCG | GATGGCGGTCAGGAGTAA |
| THRSP | NM_213577.1 | CGGTGAAGGTCAGAGCA | GGGACTTGGCACAGGAA |
| A2LD1 | XM_001231824.2 | GGAGAAGGCAAGATGGG | CTCAGCGAGGGATTAGG |
| HSP90AA1 | NM_001109785.1 | CCTGTCCTCTGGCTTTA | GTGGCATCTCCTCGGTA |
| VIT3 | ENSGALT00000002890 | GGTCCAGTTGCTTCGTGT | TAGGGTTTTCTTATTGGG |
| NCP1 | XM_004939783.1 | TGTGGCAGAAGTTATTCCC | TCACCCTCAAGACGCTCA |
| CYP17A1 | NM_001001901.2 | TCTGCTCCCTCTGCTTCAA | GACCTTCAGGCATCGCTT |
| CLU | NM_204900.1 | GTCAGTTCGGTTGGGTCTTA | CGAGGTTGGGAGTTTTGG |
| PTGS2 | NM_001167719.1 | GCGAGGAAAGCGTAAAG | CTGTTCTGACATGGGAGG |
| FABP4 | NM_204290.1 | GAGTTTGATGAGACCACAGC | TCTTTGCCATCCCACTTC |
| ADRA2A | XM_004942276.1 | CATCTTCATCGTCTGGGTCA | TCGCCTCTTGGCTATCTG |
